# Cerebro-Spinal Meningetis

**Published:** 1871-04

**Authors:** 


### CEREBRO-SPINAL MENINGITIS.

As will be seen from the proceedings of the Atlanta Academy of Medicine, our city has again been visited by *cerebro-spinal meningitis.* This visitation, as those in former years, was preceded by an epidemic of influenza, which, as heretofore, continued during the existence of the meningeal disease, and even now prevails to a limited extent in the city.

In speaking here, of influenza, we would not be understood as alluding to sporadic or accidental catarrh, but that hydra-headed monster well kown to the profession in this locality as "epidemic influenza." A disease which may assume a variety of forms in diferent individuals, and even in the same individual and during the same attack, the form or train of symptoms presented varied to a certain extent, by the tissue or organ involved, the surroundings, peculiarities, etc., of the subject attacked.

We are, from year to year, more thouroughly convinced of the correctness of our opinions formed during the epidemic of 1864, that cerebro-spinal meningitis, is a "part and parcel of this same epidemic influence, known as influenza. In other words that cerebro-spinal meningitis, as it has presented itself in this locality for the past several years, is nothing more nor less than one of the forms that this epidemic influence may present.

Those who have investigated with care, the epidemics of the past two years have not failed to note the variety of forms assumed even in the same family. In some we find the ordinary symptoms of catarrh extending to the throat larynx, etc. In others the gastro-intestinal mucous membrane seems to receive the impress, resulting in a train of symptoms resembling cholera-morbus. In others, again, the mucous membranes seem to be but little, if at all, affected, a variety of nervous symptoms being the prominent feature—as headache, earache, pleurodynia, etc., etc. In some few cases the serous membranes receive the impress, producing effusion into the articulations. Again, in other cases the head symptoms often exist for twenty-four or forty-eight hours, when pneumonia or capillary-bronchitis supervenes. But the most troublesome, and certainly the most fatal form is where the meninges of the brain and spinal cord (?) receive the first shock or impress of this epidemic influence, and most frequently occurs, following some one of the varied train of symptoms above aluded to.

We do not set ourselves up as authority upon this subject, nor do we propose to insist upon our views, but would be permitted again to say that in our judgment cerebro-spinal meningitis, as it has existed in this locality for the past nine years, is one of the forms or accidents of "epidemic influenza."

